# Neuroprotective potency of mangiferin against 3-nitropropionic acid induced Huntington’s disease-like symptoms in rats: possible antioxidant and anti-inflammatory mechanisms

**DOI:** 10.3389/fphar.2023.1189957

**Published:** 2023-07-13

**Authors:** Pei Teng Lum, Mahendran Sekar, Lay Jing Seow, Mohd Farooq Shaikh, Alina Arulsamy, Thaarvena Retinasamy, Siew Hua Gan, Charles Gnanaraj, Norhaizan Mohd Esa, Gobinath Ramachawolran, Vetriselvan Subramaniyan, Suresh V. Chinni, Yuan Seng Wu

**Affiliations:** ^1^ Department of Pharmaceutical Chemistry, Faculty of Pharmacy and Health Sciences, Royal College of Medicine Perak, Universiti Kuala Lumpur, Ipoh, Malaysia; ^2^ School of Pharmacy, Monash University Malaysia, Subang Jaya, Selangor, Malaysia; ^3^ School of Dentistry and Medical Sciences, Charles Sturt University, Orange, NSW, Australia; ^4^ Neuropharmacology Research Laboratory, Jeffrey Cheah School of Medicine and Health Sciences, Monash University Malaysia, Subang Jaya, Selangor, Malaysia; ^5^ Faculty of Pharmacy and Health Sciences, Royal College of Medicine Perak, Universiti Kuala Lumpur, Ipoh, Perak, Malaysia; ^6^ Department of Nutrition, Faculty of Medicine and Health Sciences, Universiti Putra Malaysia, Serdang, Selangor, Malaysia; ^7^ Department of Foundation, RCSI & UCD Malaysia Campus, Georgetown, Penang, Malaysia; ^8^ Jeffrey Cheah School of Medicine and Health Sciences, Monash University Malaysia, Subang Jaya, Selangor, Malaysia; ^9^ Center for Transdisciplinary Research, Department of Pharmacology, Saveetha Dental College, Saveetha Institute of Medical and Technical Science, Saveetha University, Chennai, India; ^10^ Department of Biochemistry, Faculty of Medicine, Bioscience, and Nursing, MAHSA University, Jenjarom, Selangor, Malaysia; ^11^ Department of Periodontics, Saveetha Dental College and Hospitals, Saveetha Institute of Medical and Technical Sciences, Saveetha University, Chennai, India; ^12^ School of Medical and Life Sciences, Sunway University, Subang Jaya, Selangor, Malaysia

**Keywords:** 3-nitropropionic acid, Huntington’s disease, mangiferin, natural product, neuroprotective

## Abstract

Huntington’s disease (HD), a neurodegenerative disease, normally starts in the prime of adult life, followed by a gradual occurrence of psychiatric disturbances, cognitive and motor dysfunction. The daily performances and life quality of HD patients have been severely interfered by these clinical signs and symptoms until the last stage of neuronal cell death. To the best of our knowledge, no treatment is available to completely mitigate the progression of HD. Mangiferin, a naturally occurring potent glucoxilxanthone, is mainly isolated from the *Mangifera indica* plant. Considerable studies have confirmed the medicinal benefits of mangiferin against memory and cognitive impairment in neurodegenerative experimental models such as Alzheimer’s and Parkinson’s diseases. Therefore, this study aims to evaluate the neuroprotective effect of mangiferin against 3-nitropropionic acid (3-NP) induced HD in rat models. Adult Wistar rats (*n* = 32) were randomly allocated equally into four groups of eight rats each: normal control (Group I), disease control (Group II) and two treatment groups (Group III and Group IV). Treatment with mangiferin (10 and 20 mg/kg, p. o.) was given for 14 days, whereas 3-NP (15 mg/kg, i. p.) was given for 7 days to induce HD-like symptoms in rats. Rats were assessed for cognitive functions and motor coordination using open field test (OFT), novel object recognition (NOR) test, neurological assessment, rotarod and grip strength tests. Biochemical parameters such as oxidative stress markers and pro-inflammatory markers in brain hippocampus, striatum and cortex regions were evaluated. Histopathological study on brain tissue was also conducted using hematoxylin and eosin (H&E) staining. 3-NP triggered anxiety, decreased recognition memory, reduced locomotor activity, lower neurological scoring, declined rotarod performance and grip strength were alleviated by mangiferin treatment. Further, a significant depletion in brain malondialdehyde (MDA) level, an increase in reduced glutathione (GSH) level, succinate dehydrogenase (SDH), superoxide dismutase (SOD) and catalase (CAT) activities, and a decrease in tumor necrosis factor-alpha (TNF-α), interleukin-1 beta (IL-1β) and interleukin-6 (IL-6) levels were observed in mangiferin treated groups. Mangiferin also mitigated 3-NP induced histopathological alteration in the brain hippocampus, striatum and cortex sections. It could be inferred that mangiferin protects the brain against oxidative damage and neuroinflammation, notably via antioxidant and anti-inflammatory activities. Mangiferin, which has a good safety profile, may be an alternate treatment option for treating HD and other neurodegenerative disorders. The results of the current research of mangiferin will open up new avenues for the development of safe and effective therapeutic agents in diminishing HD.

## Graphical Abstract

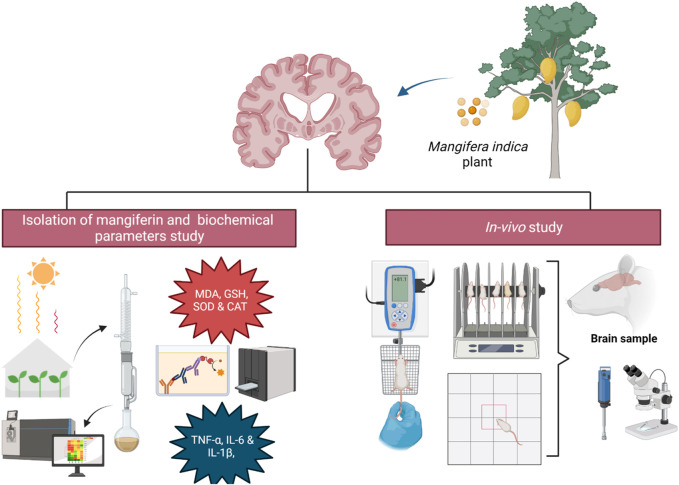

## 1 Introduction

Huntingtin (Htt) is a protein formed by more than 3100 amino acids, encoded by a gene located at chromosome 4 (Gil and Rego, 2008). Huntington’s disease (HD) is a fatal and progressive neurodegenerative disease associated with a pathogenic expansion of cytosine-adenine-guanine (CAG) trinucleotide repeats in exon one of the huntingtin gene (Htt) (Roos, 2010). HD was first recognized and clinically described via a published article, “On Chorea”, by George Huntington in 1872 (Huntington, 2003; Kay et al., 2017). Despite discerning common HD genetic origins, disease progression tends to vary based on an individual’s mutation rates and diagnostic stigma. Gene mutation is the main causative of HD discovered in 1933 (Kay et al., 2017; Vicente et al., 2021). In this context, mutant Htt (mHtt), a mutated functional protein carrying abnormal and elongated polyglutamine (polyQ), is the main culprit contributing to the pathophysiology of HD (Gil and Rego, 2008). In Malaysia, the first HD case of a man aged 40 diagnosed with 2 years of cognitive dysfunction and 10 years of movement disturbance was documented in 1994 (Lee et al., 1994). Along with this, a nationwide HD registry was first established at the University of Malaya Medical Centre in 1995. The data from meta-analyses have reported a global prevalence of approximately 2.7 in 100,000 HD, with the lowest incidence seen among Asians and the highest in Western populations (Bashir, 2019). However, the overall incidence of HD worldwide remains unclear because epidemiological evidence from Asia and African populations is limited to only clinical reviews and case studies (Bates et al., 2015).

**FIGURE 1:**
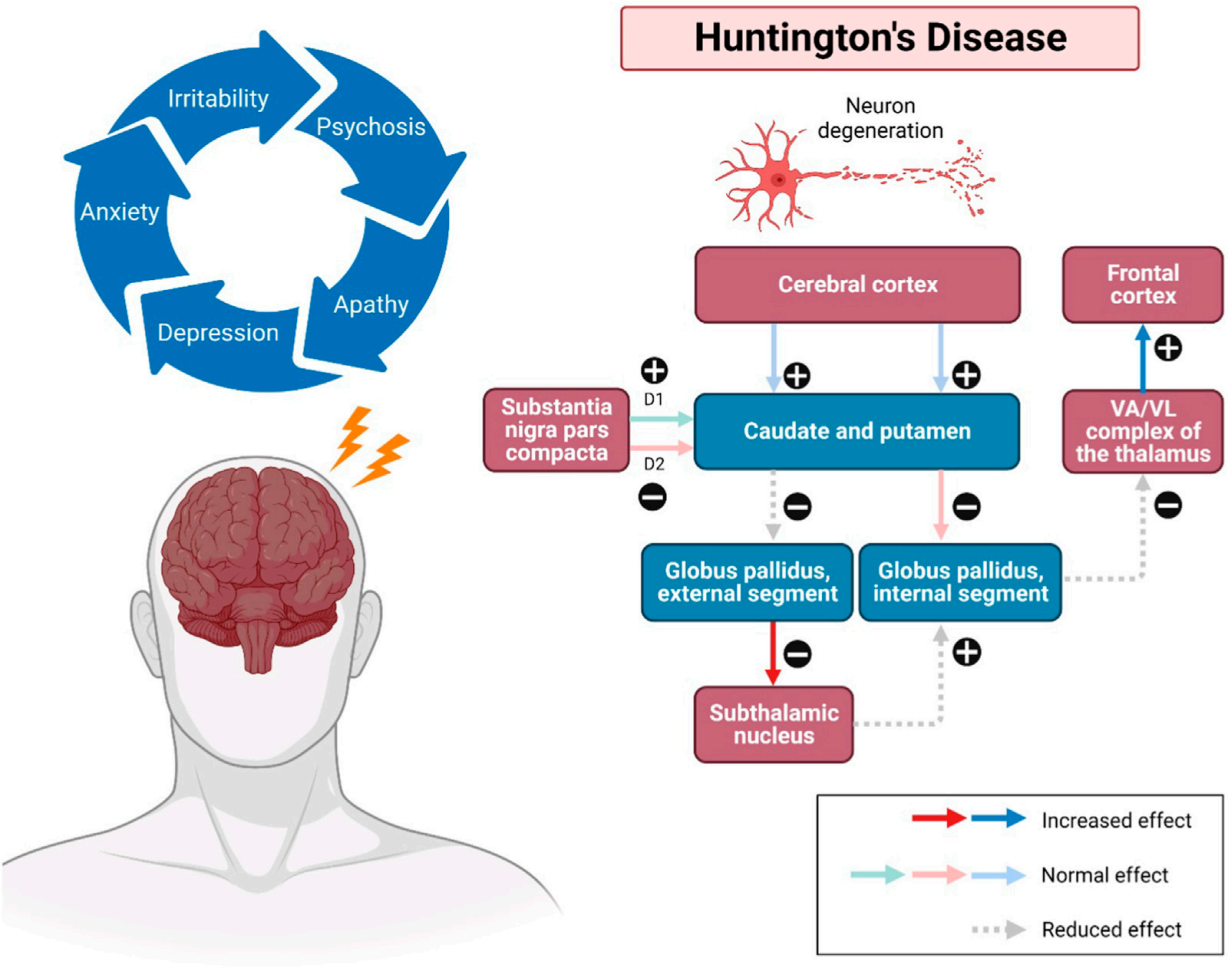
Pathways involved in Huntington’s disease (HD). Impacted striatal neurons, leading to atrophy of the cerebral cortex, subsequently the caudate and putamen, as well as the globus pallidus (internal and external). The external globus pallidus will receive fewer neurotransmitters, causing false signals that allow more neurotransmitters to be released to the subthalamic nuclei. Additionally, neurons can also be disintegrated in the regions of the hypothalamus, thalamus, and zona reticulata of the substantia nigra. Substance P, enkephalin, and dynorphin are found in susceptible striatal neurons, which mainly innervate the substantia nigra and globus pallidus. The upregulation and modulatory effects may cause a sequence of neurodegenerative events, including overstimulation of the motor cortex, thus showing the progression of the disease. Abbreviations: VA, ventral anterior nucleus; VL, ventral lateral nucleus.

The onset of HD occurs between 30 and 50 years (mean survival, 15–20 years). HD occurring before 21 years of age is deemed a juvenile HD, while the other extreme, “late-onset”, occurs after 60 years old (Gil and Rego, 2008). The clinical features of HD predominantly include progressive psychiatric, cognitive disturbances and motor dysfunction (Figure 1). The lifestyle of HD patients is disturbed by irritability, aggression, depression, anxiety, low self-esteem, apathy and subsequent by psychosis (Ross et al., 2014). Along with these risk factors, therefore, suicidality is also considered a serious negative impact that has been extensively investigated among HD patients over the last decades. Numerous studies have revealed that HD patients tend to have suicidal behaviour and need to be closely monitored (Freitas and Valadas, 2021). Besides, their memory gets impaired, and language is comparatively spared. Their psychomotor processes such as planning, cognitive flexibility and problem-solving are getting severely impaired (Wheelock et al., 2003). In addition, altered motor performances progressively trigger difficulties in their daily life. Consequently, these clinical signs and symptoms have severely interfered with the mental well-being, level of independence, cognition performances, and life quality of HD patients until the last stage of neuronal cell death (Maffi et al., 2022). However, there are no promising treatments for long-term disease modification and complete attenuation of HD. In the recent status of HD drug therapies, only tetrabenazine is approved by Food and Drug Administration (FDA) to treat chorea and other HD-related motor symptoms by modulating dopamine receptors (Wang et al., 2010; de Tommaso et al., 2011; Kumar et al., 2020) Available therapeutics of HD mainly target Htt aggregation, transcriptional dysregulation, mitochondrial dysfunction, excitotoxicity via dopamine and glutamate pathways, caspase and stem cell transplants (Kumar et al., 2020). Strikingly, only symptomatic prevention and treatments may be beneficial to some individuals for their daily performances. Therefore, it is of paramount crucial to identify the therapeutic benefits of novel natural compounds as alternative treatment approaches for HD in future.

To date, various *in vitro* and *in vivo* HD models have been established. Among existing animal models of HD, 3-NP induced model has been recognized as the most recent and reliable study model for HD, owing to the spontaneous, bilateral and specific striatal lesions produced by systemic treatment of 3-NP in animal brains (Brouillet, 2014). 3-NP, a metabolite of 3-nitropropanol, is a mycotoxin naturally produced by a variety of fungal species such as *Astragalus arthrinium* and *Aspergillus flavus*, and also present in certain plant species such as *Indigofera endecapylla* and leguminous plants (Túnez et al., 2010). Owing to the permeability of 3-NP through the blood-brain barrier (BBB), it is well-known as a mitochondrial neurotoxin that can induce a downstream process of neurodegeneration via the inhibition of SDH (Danduga et al., 2018). In 3-NP intoxicated rodent models, 3-NP was shown to (1) irreversibly inhibits SDH activity, (2) subsequently blocks the electrons transport chain and results in (3) a failure of energy metabolism in the brain, followed by (4) mitochondrial dysfunction (Shivasharan et al., 2013; Gao et al., 2015). These events provide an environment with (1) depleted endogenous antioxidant system, and (2) an elevation of intracellular oxidative-nitrosative stress in the brain, thereby promoting (3) excitotoxicity due to increased glutamate and cytosolic calcium level attributed to the blockage of the receptor ion channel by free radicals (Sandhir et al., 2010; Mehan et al., 2018). In this direction, neuroinflammatory responses via nuclear factor kappa B (NF-κB) activation are also induced by oxidative stress pathways (Singh et al., 2015). In the neuropathology of HD, progressive neuronal degeneration is mainly observed in the brain regions of striatum and cerebral cortex (Nopoulos, 2022). Other parts of brain such as hippocampus is also affected (Harris et al., 2019). Therefore, 3-NP induced specific striatal lesions in rats, mimicking characteristics in HD, making 3-NP a promising experimental model to enrich our understanding of HD pathophysiology.

**FIGURE 2:**
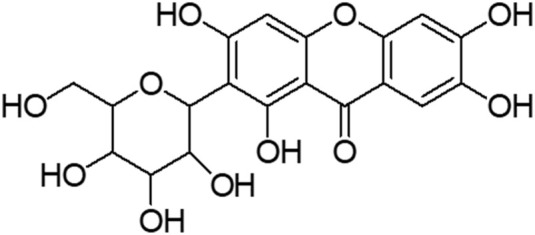
Structure of mangiferin.

Mangiferin (molecular formula: C_19_H_18_O_11_; molecular weight: 422 g/mol; structural name: 1,3,6,7-tetrahydroxyxanthone C2-β-D-glucoside) (Figure 2) is a naturally occurring pharmacologically glucoxilxanthone which being extensively investigated for its biological and therapeutic potentials. Accordingly, Walia et al. (2020) reported that mangiferin is widely present in 96 species, 28 genera and 19 families of angiospermic plants. Mangiferin has also been found in certain monocots and ferns such as *Acystopteris* sp., *Asplenium adiantum-nigrum*, *Cystopteris* sp., *Davallia subsolida*, *Gymnocarpium* sp., *Trichomanes reniforme* and *Woodsia* sp., as well as in young leaves of *Coffea pseudozanguebariae* (Matkowski et al., 2013; Sekar, 2015; Jyotshna et al., 2016). However, *Mangifera indica* (Family: Anacardiaceae), commonly known as Mango which is abundantly available in Malaysia, is the primitive and chief source of mangiferin. Despite of the exact mechanism of mangiferin absorbed through blood−brain barrier (BBB) is unknown, an intense effort has been devoted to evaluating the therapeutic potential of mangiferin against neurological disorders (Feng et al., 2019; Walia et al., 2020; Liu et al., 2021). Accumulating studies implicate that mangiferin offers neuroprotection to the CNS against mitochondrial dysfunction, oxidative stress, cellular apoptosis, and neuroinflammation. It also improves the memory loss and declined cognition of rats under memory impairment *in vivo* model (Lum et al., 2020). Building on the information from the literature, mangiferin appears to be a promising agent against HD. Furthermore, understanding the neurotherapeutic efficacy of mangiferin with its underlying mechanisms is of great significance. Owing to a paucity of research of mangiferin on HD, the present study was conducted to evaluate the neuroprotective efficacy of mangiferin against 3-NP induced HD in rats.

## 2 Materials and methods

### 2.1 Isolation of mangiferin from *mangifera indica*

Extraction and isolation of mangiferin were performed following the Soxhlet method according to the previous procedures by Sekar et al. (2014). Fresh mango leaves were shade-dried, powdered and defatted with petroleum ether, followed by the extraction process with ethanol under continuous reflux in the Soxhlet apparatus at 40°C–50°C for 20 h. After rotary evaporation, the concentrated extract was kept overnight at room temperature. Solid precipitation was separated, filtered and washed repeatedly with petroleum ether till a yellowish powdered mangiferin was acquired. The powder was then repeatedly crystallised in aqueous ethyl acetate and methanol, and pale-yellow needle-shaped mangifeirn crystal (1,3,6,7-tetrahydroxyxanthone-C2-β-D-glucoside) was yielded. The purity of isolated mangiferin was identified by high performance thin layer chromatography (HPTLC) using the solvent system of ethyl acetate: methanol: water: formic acid (6:2:1:1, R_f_ = 0.76) with authentic sample.

### 2.2 Animals

The study was conducted in accordance with the Animal Research Review Panel (ARRP) guidelines on wildlife research for the use and care of laboratory animals. The experiment protocols were approved by Monash University Malaysia Animal Ethics Committee (MUM AEC) (27795). 32 mixed-gender Wistar rats, aged 4–5 months, were procured from the Institutional Animal House Unit. All rats were housed under standard laboratory conditions, maintained at a constant temperature of 20°C ± 2°C and a 12-h light−dark cycle with free access to food and water.

### 2.3 Experimental protocols

In this study, the entire treatment protocol was conducted for 14 days. Rats were randomly categorized equally into four groups of eight rats (*n* = 8) in each. The treatment was given as follows:

Group I: Normal control (Saline, p. o.).

Group II: Disease control [3-NP (15 mg/kg/day, p. o.)].

Group III: Mangiferin (10 mg/kg/day, p. o.) + 3-NP (15 mg/kg/day, i. p.).

Group IV: Mangiferin (20 mg/kg/day, p. o.) + 3-NP (15 mg/kg/day, i. p.).

**FIGURE 3:**
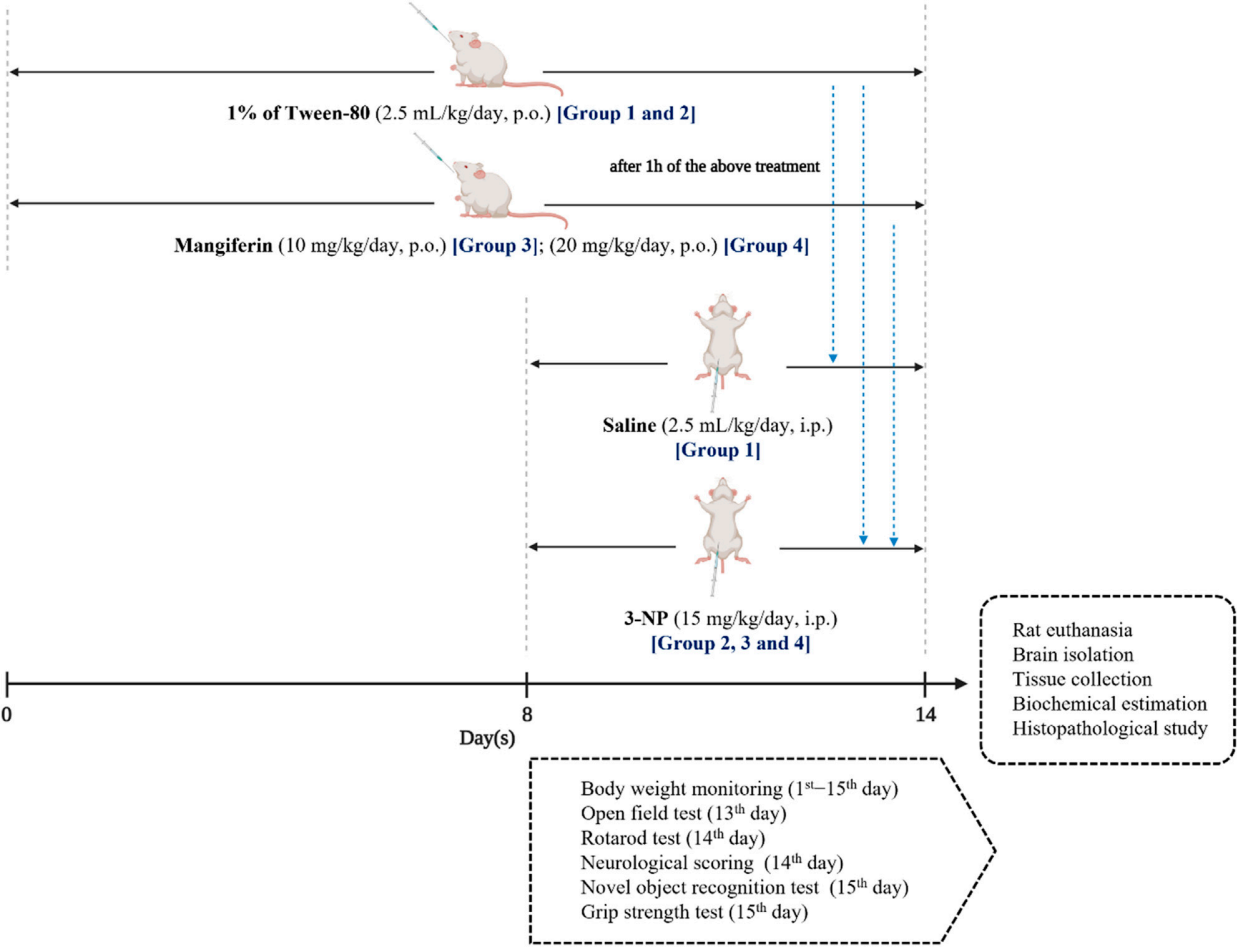
Experimental design.

One percentage Tween-80 was given by oral gavage to Groups I and II, while mangiferin was suspended in distilled water using 1% Tween-80 (v/v) and administered orally at the doses of 10 and 20 mg/kg/day to Group III and IV, respectively for 14 days. From eighth day to 14th day, 1 h after the above treatments, buffered saline (pH 7.4) was given intraperitoneally (i. p.) to Group I, while 3-NP was diluted in with saline and administered by an intraperitoneal route at a dose of 15 mg/kg/day to Group II, III and IV to induce HD-like symptoms. The experimental design is presented in Figure 3.

### 2.4 Body weight monitoring

The body weight of rats was measured on a daily basis prior to the drug administration from the 1st to 15th day throughout the experiment. The percentage of changes in body weights was calculated in comparison to the rats’ initial body weights recorded, given by:

$$Body\;weight\;change\;\left(\%\right):\frac{\left(Wt_{15}-Wt_{0}\right)}{Wt_{0}}\times 100$$

where *Wt*_0_ and *Wt*_15_ refer to the rats initial body weight and at 15th day of the experiment, respectively.

### 2.5 Behavioral assessment

All the behavioral assessments were conducted during the light portion between 10:00 a.m. and 4:00 p.m. to avoid circadian influences. 3 h after 3-NP administration, rats were assessed for OFT on the 13th day, while neurological scoring and rotarod test were conducted on the 14th day. NOR and grip strength test were performed before rat euthanasia on the 15th day of the experiment (Figure 3). Between each test session, the apparatus were cleaned with 75% ethanol solution to avoid the effect of olfactory cues among rats. Prior to behavioral assessments, rats were placed at the behavioral room to acclimate the new environment for 30 min.

#### 2.5.1 Open field test

OFT was used to assess locomotor activity and anxiety-like behaviour as previously described by Wang L. et al. (2017). The open field apparatus was equipped with a black acrylic square box (70.8 cm × 70.8 cm) at the height of 36 cm. Each rat was placed at the center of the open arena. Total distance travelled (indicator of locomotor activity) and time spent in the center of the arena (indicator of anxiety) were recorded for 5 min and analyzed using video tracking software EthoVision XT version 15.

#### 2.5.2 Novel object recognition test

NOR test was employed to evaluate the recognition memory and learning of rats following the previous protocol by Lueptow (2017) and Antunes and Biala (2012). The experimental set-up was mainly equipped with a black acrylic square box (70.8 cm × 70.8 cm) at the height of 36 cm. The test was conducted in three phases, as depicted in Supplementary Figure S1: habituation, familiarization and testing phase. In the habituation phase, an individual rat was placed at the center of the arena and allowed for free exploration without objects for 5 min. For familiarization, two identical objects (objects A1 and A2) were affixed at two corners equidistantly from the respective arena walls and from each other. Each rat was placed at the center of the arena with its tail facing the two identical objects and allowed to begin the objects’ exploration for 10 min by touching or sniffing the objects. In the testing phase, the object A2 was replaced with a novel object B which is different in colour, shape and size. The individual rat was allowed to explore familiar (A1) and novel (B) objects for 10 min. The time spent exploring familiar object A1 and novel object B were recorded and analyzed using video tracking software EthoVision XT version 15. The recognition index is defined as:

$$Recognition\;index\;\left(\%\right):\frac{T_{B}}{\left(T_{A}+T_{B}\right)}\times 100$$

where *T*_*A*_ and *T*_*B*_ refer to the time spent to explore familiar object A1 and novel object B, respectively.

#### 2.5.3 Neurological assessment

Neurological scoring was conducted to assess the motor impairment of rats through the specific observational tests designed to evaluate their ambulatory movements. In this study, a rapid neurological scoring protocol (Table 1) was used. It was adopted from a score sheet of neurobehavioral phenotype assessment developed by Ditzler et al. (2003), revised neurobehavioral severity scale (NSS-R) for rodents by Yarnell et al. (2016) and neurological scoring described by Danduga et al. (2018). Each rat was scored for a series of neurobehavioral and physical assessments simultaneously in a square box for 2 min.

**TABLE 1:** Neurological scoring protocol of rat HD model.

Neurobehavioral assessment	
Movement analysis	Rats were evaluated for their normal OR slow OR in-coordinated movement OR hind limb paralysis OR incapacity to move
Grooming	Rats were observed for the normal (slow/occasional) OR abnormal (none/excessive) grooming activity
Tail raise test	Rats were suspended by the tail for 15 s and observed for the normal (extending), partial (twisting) OR no limbs reflexes
Physical assessment	
Palpebral closure	Rats were observed for the wide-open OR flattened, swollen lids or squinty eyes
Piloerection	Rats were evaluated for the normal (smooth fur) OR abnormal (erected fur) fur condition on the back
Body position	Rats were evaluated for the normal (elongated) OR abnormal (hunched or rounded) body position while moving
Tail position	Rats were observed for the normal (horizontally extended) OR abnormal (dragging) tail position

#### 2.5.4 Rotarod test

The motor coordination of rats was evaluated using a rotarod apparatus following the previous protocol by Danduga et al. (2018). The rotarod consists of an elevated and horizontal rod with 7 cm in diameter that operates along the longitudinal axis. Prior to the main test, rats were trained for two consecutive days. Each rat was first placed and habituated on the stationary rod for 10 s, followed by three test trials on the rotating rod at a constant speed of 25 rpm with intervals of 5 min. The latency of the rats to fall off the rotating rod was recorded. The cut-off time of 180 s was given. The average latency to fall off from three trials was calculated as follows:

$$Latency\;of\;fall\;off\;\left(s\right):\frac{F_{T1}+F_{T2}+F_{T3}}{3}$$

where *F*_*T1*_, *F*_*T2*_ and *F*_*T3*_ refer to the latency of the rats to fall off the rotating rod at trial 1, trial 2, and trial 3, respectively.

#### 2.5.5 Grip strength test

A grip strength test was performed to assess the grip strength of rats according to the previous protocol applied by Forgione et al. (2017) and Jackson et al. (2015). The apparatus set-up consists of a wire mesh (41.5 cm × 25 cm) with an empty rodent cage. Habituation of rats on this test was performed for two consecutive days before the main testing session. Each rat was placed on top of the wire mesh at the height of approximately 19 cm above the rodent cage. It was then shaken gently and horizontally three times to induce the rat to grasp onto wire mesh with all paws. The hanging time was recorded once the wire mesh was inverted till the rat falls off to the home cage. The experimental procedure is illustrated in Supplementary Figure S2. A total of three trials were conducted, and a cut-off time of 90 s was taken. The average latency of grip loss was calculated and defined as:

$$Latency\;of\;grip\;loss\;\left(s\right):\frac{G_{T1}+G_{T2}+G_{T3}}{3}$$

where *G*_*T1*_, *G*_*T2*_ and *G*_*T3*_ refer to the latency of grip loss at trial 1, trial 2, and trial 3, respectively.

### 2.6 Brain tissue collection

Prior to euthanasia, rats were anesthetized via intraperitoneal injection of a mixture of ketamine (75 mg/kg) and xylazine (10 mg/kg). The rats were transcardially perfused with 0.9% ice-cold saline and the whole brain was isolated. The hippocampus, striatum and cortex were dissected using sterilized surgical blade and ophthalmic forceps. Sectioned brain parts were then kept immediately on dry ice before being stored in a deep freezer at −80°C. To prepare brain homogenate, the brain samples were homogenized in ice-cold phosphate buffered saline (PBS) (10% w/v) on ice. Homogenized brain samples were then centrifuged at 12,000 × g for 15 min at 4°C. The supernatant was collected and used for the following biochemical analysis.

### 2.7 Biochemical estimation

#### 2.7.1 Determination of oxidative stress markers

To evaluate the antioxidant effects of mangiferin on HD-induced rats, oxidative stress parameters such as SDH activity, MDA and GSH level, SOD and CAT activities in the hippocampus, striatum and cortex of rat brain were determined. The level of SDH was determined using an ELISA kit based on the Sandwich-ELISA principle. The extent of lipid peroxidation was quantified by measuring the MDA concentration using an ELISA kit based on the Competitive-ELISA principle. The level of GSH and CAT activity were determined by colorimetric method using a GSH and the CAT Activity Colorimetric Assay kit, respectively. The SOD activity was determined by the hydroxylamine method using the T-SOD Activity Assay kit. All assay was performed according to the standard protocols provided by Elabscience^®^.

#### 2.7.2 Determination of pro-inflammatory markers

To evaluate the potential of mangiferin in reducing the inflammation on HD-induced rats, pro-inflammatory markers, including TNF-α, IL-6, and IL-1β, were determined using ELISA kits based on the Sandwich-ELISA principle. The assay was performed based on the standard protocols provided by Elabscience^®^.

### 2.8 Histopathological study

Rats were transcardially perfused with 4% Paraformaldehyde (PFA). The isolated brains were post-fixed in 4% PFA solution for 24 h. All fixed brain tissues were dehydrated in a serial dilution graded alcohol, followed by a clearing process with xylene and impregnation with liquid paraffin. The processed tissues were then embedded in paraffin wax and sectioned into 5 µm thickness. The slides were stained with hematoxylin and eosin (H&E) and observed under a light microscope with a magnification of ×100.

### 2.9 Statistical analysis

All data were statistically analysed using GraphPad Prism version 9.3.1 and expressed as mean ± standard error of mean (SEM). Body weight changes and all behavioural performances were analysed using a one-way analysis of variance (ANOVA). Post hoc testing was performed using Turkey’s method for multiple comparison among groups. Kruskal Wallis non-parametric analysis of variance was used for neurological scoring, all parameters of oxidative stress and inflammatory markers followed by Dunn’s multiple comparisons test. In this study, *p* values <0.05 was considered statistically significant for all analysis.

## 3 Results

### 3.1 Effect of mangiferin on body weight

**FIGURE 4:**
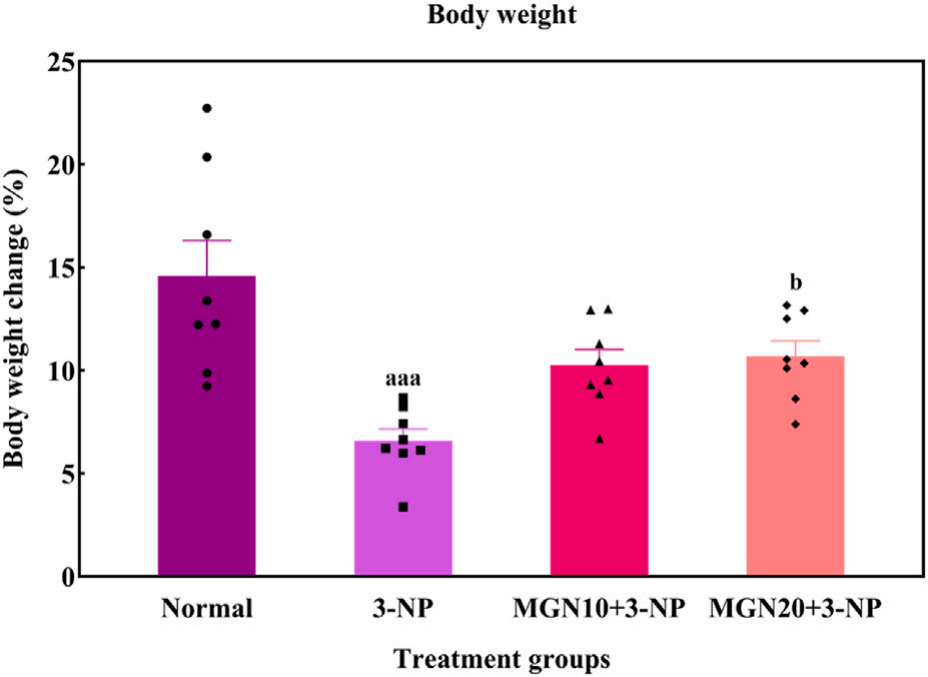
Effect of mangiferin on body weight changes. Data represents mean ± SEM, *n* = 8 per group. ^aaa^ *p* < 0.001 compared to normal control group; ^b^ *p* < 0.05 compared to 3-NP alone treated group.

In this study, the body weights of all rats were gradually increased throughout the treatment process (Figure 4). However, rats with 3-NP treatment (15 mg/kg, i. p.) for 7 days showed a reduced percentage of body weight gain as compared to normal control rats (*p* < 0.001). Importantly, mangiferin treatment (20 mg/kg, p. o.) along with 3-NP greatly improved the body weight gain of rats as compared to 3-NP alone treated group (*p* < 0.05). The mangiferin treated group at a dose of 10 mg/kg also showed the improvement in the body weight gain but it was not significant as compared to the 3-NP alone treated group (*p* > 0.05).

### 3.2 Effect of mangiferin on anxiety and cognitive functions

#### 3.2.1 Anxiety related behaviour

**FIGURE 5:**
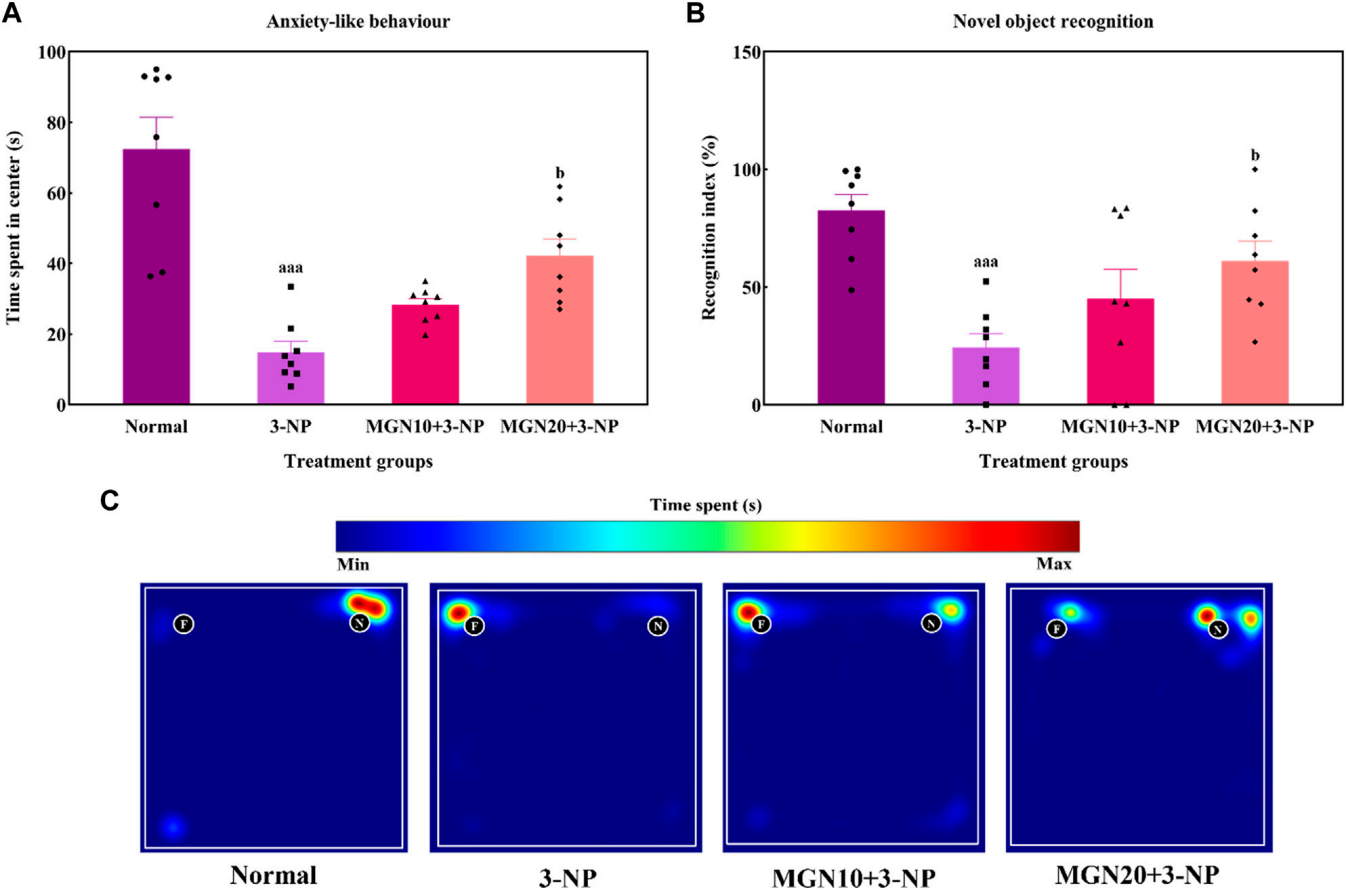
Effect of mangiferin on **(A)** anxiety-like behaviour and **(B)** NOR. **(C)** Representative heat map visualisation in NOR test, indicating time spent and location near the objects. Data represents mean ± SEM, *n* = 8 per group. ^aaa^ *p* < 0.001 compared to normal control group; ^b^ *p* < 0.05 compared to 3-NP alone treated group.

Anxiety related behaviour of rats was evaluated in terms of the time spent in center arena in the OFT. Rats treated with 3-NP significantly spent less time in center arena than normal control rats, indicating more anxiety was triggered by 3-NP (*p* < 0.001) (Figure 5A). In contrast, less anxious behaviour evident by more time spent in center was observed in mangiferin (20 mg/kg, p. o.) treated groups as compared to 3-NP alone treated group (*p* < 0.05). Mangiferin treatment (10 mg/kg, p. o.) did not produce any significant effect on anxiety-like behaviour as compared to 3-NP alone treated group (*p* > 0.05).

#### 3.2.2 Novel object recognition

In the NOR test, the ability of rats to differentiate between a familiar and novel object is denoted by a recognition index. The recognition index was significantly reduced by 3-NP treatment (15 mg/kg, i. p.) in comparison to the normal control group (*p* < 0.001) (Figure 5B). Indeed, this trend could be seen in the heat map with 3-NP treated rats showing less exploratory time near the novel object (Figure 5C). Furthermore, mangiferin (20 mg/kg, p. o.) treated rats demonstrated a higher preference for exploration of the novel object along with an increased recognition index in comparison to 3-NP alone treated rats (*p* < 0.05). However, the recognition memory was not significantly improved by mangiferin oral treatment at a dose of 10 mg/kg compared to the 3-NP alone treated group (*p* > 0.05).

### 3.3 Effect of mangiferin on motor coordination

#### 3.3.1 Locomotor activity

**FIGURE 6:**
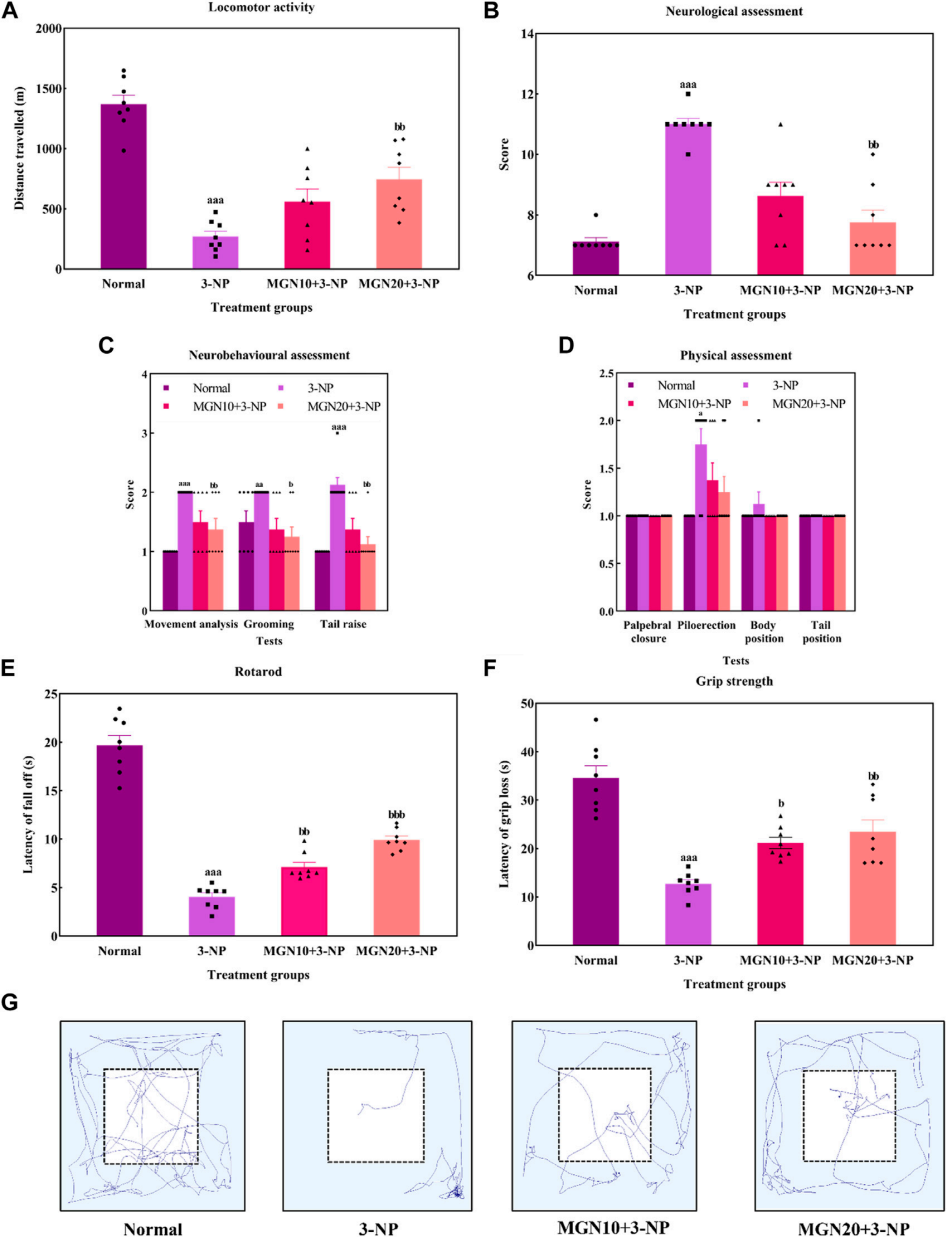
Effect of mangiferin on **(A)** locomotor activity, **(B)** total neurological scoring, **(C)** neurobehavioural and **(D)** physical assessments, **(E)** rotarod, and **(F)** Grip strength. **(G)** Representative track visualisation of rats in open field arena. Data represents mean ± SEM, *n* = 8 per group. ^a^ *p* < 0.05, ^aa^ *p* < 0.01, ^aa^ *p* < 0.001 compared to normal control group; ^b^ *p* < 0.05, ^bb^ *p* < 0.01 and ^bb^ *p* < 0.01 compared to 3-NP alone treated group.

General locomotor activity is an animal’s spontaneous and physical motion from one place to another (Lever et al., 2017). 3-NP administration (15 mg/kg, i. p.) significantly diminished the locomotor activity of rats with the lowest distance travelled observed in the 3-NP treated group when compared to the normal control group (*p* < 0.001) (Figure 6A). The finding was confirmed by the trajectory of rats in which 3-NP treated rats moved less than normal control rats (Figure 6G). On the contrary, the impairment in locomotor activity was markedly mitigated by a higher dose of mangiferin at 20 mg/kg compared to the 3-NP alone treated group (*p* < 0.05). However, there was no significant difference in locomotor activity of rats between the lower dose of mangiferin (10 mg/kg, p. o.) treated group and the 3-NP alone treated group (*p* > 0.05).

#### 3.3.2 Neurological scoring

In this study, the neurological assessment was conducted based on a scoring system using the Likert scale whereby normal is assigned as “1” whereas abnormal is assigned as “2”. Apparently, a higher neurological score was gained by the 3-NP treated rats in comparison to normal control rats (*p* < 0.001) (Figure 6B). In fact, oral administration of mangiferin at a dose of 20 mg/kg considerably improved neurological behaviours as a lower neurological score was achieved in comparison to 3-NP alone treated group (*p* < 0.01). Nevertheless, there was no significant improvement in the neurological score in the case of oral mangiferin treatment at 10 mg/kg dose compared to the 3-NP alone treated group (*p* > 0.05).

As compared to the normal control group, administration of 3-NP (15 mg/kg, i. p.) significantly caused abnormal neurobehaviors in which higher scores were obtained for movement analysis (*p* < 0.001), grooming (*p* < 0.01) and tail raise assessments (*p* < 0.001) (Figure 6C). On treatment with mangiferin (20 mg/kg, p. o.), rats exhibited a remarkable decrease in scores for movement analysis (*p* < 0.01), grooming (*p* < 0.05) and tail raise tests (*p* < 0.01) when compared to 3-NP alone treated group. Oral administration of mangiferin at 10 mg/kg dose did not significantly improve the scores compared to the 3-NP alone treated group (*p* > 0.05).

For physical assessment, both 3-NP administration (15 mg/kg, i. p.) and mangiferin treatment (10 and 20 mg/kg, p. o.) did not affect the palpebral closure, body and tail position of rats (*p* > 0.05) (Figure 6D). On the other hand, rats intraperitoneally treated with 3-NP gained higher scores on piloerection as compared to the normal control group (*p* < 0.05), whereas mangiferin oral administration at both doses (10 and 20 mg/kg, p. o.) did not markedly alter the neurological score of piloerections as compared to 3-NP alone treated group (*p* > 0.05).

#### 3.3.3 Rotarod

Rotarod test was performed to assess the body balance and motor coordination of rats. Rats given with 3-NP treatment (15 mg/kg, i. p.) showed a remarkable decline in motor performances with evidence of decreased latency to fall off from the rotarod when compared to the normal control group (*p* < 0.001) (Figure 6E). In both treatment groups, rats exhibited longer latency to fall off from rotarod. The findings showed that mangiferin treatments (10 and 20 mg/kg, p. o.) ahead of 3-NP greatly restored the motor coordination of rats in a dose-dependent manner when compared to 3-NP alone treated group (*p* < 0.01 and *p* < 0.001).

#### 3.3.4 Grip strength

The grip strength of rats was evaluated as latency of grip loss using a wire mesh. On the treatment of 3-NP (15 mg/kg, i. p.), shorter hanging latency was observed as compared to the normal control group (*p* < 0.001) (Figure 6F). This result indicates that 3-NP significantly induced the rats’ grip strength loss. In contrast, oral administration of mangiferin at both doses of 10 and 20 mg/kg dose-dependently improved grip strength, which was evident from increased grip loss latency as compared to the 3-NP alone treated group (*p* < 0.05 and *p* < 0.01).

### 3.4 Effect of mangiferin on oxidative stress markers

#### 3.4.1 SDH

**FIGURE 7:**
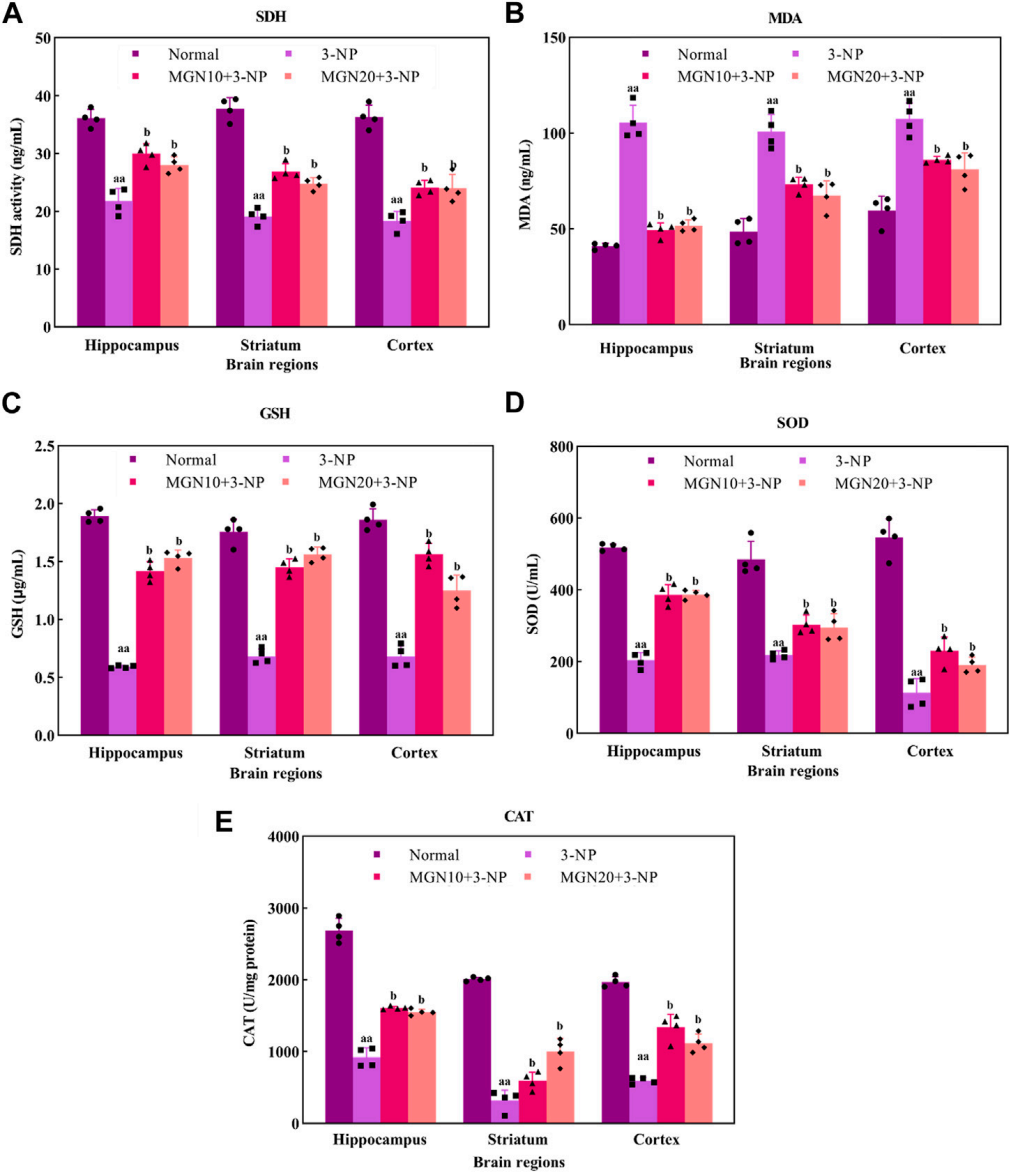
Effect of mangiferin on oxidative stress markers: **(A)** SDH activity, **(B)** MDA, **(C)** GSH level, **(D)** SOD, and **(E)** CAT activities. Data represents mean ± SEM, *n* = 4 per group. ^aa^ *p* < 0.01 compared to normal control group; ^b^ *p* < 0.05 compared to 3-NP alone treated group.

SDH, mitochondrial complex II, is a key enzyme involved in the oxidation of succinate to fumarate in the Krebs cycle ETC (Rustin et al., 2002). Administration of 3-NP (15 mg/kg, i. p.) for 7 days substantially decreased SDH activity in the brain regions of the hippocampus, striatum and cortex as compared to the normal control group (*p* < 0.01) (Figure 7A). Surprisingly, mangiferin treatments (10 and 20 mg/kg, p. o.) for 14 days respectively restored 3-NP induced depletion of SDH activity in all three brain regions as compared to the 3-NP alone treated group (*p* < 0.05).

#### 3.4.2 MDA

MDA is one of the secondary products produced from the decomposition of polyunsaturated fatty acids (PUFAs). It has been commonly used as an oxidative stress marker for lipid peroxidation through its reaction with thiobarbituric acid (TBA) (Ayala et al., 2014). The level of MDA in the hippocampus, striatum and cortex was significantly higher in 3-NP treated group compared to the normal control group (*p* < 0.01) (Figure 7B). However, mangiferin-treated groups showed a lower level of MDA in these brain regions than in 3-NP alone treated group (*p* < 0.05). Oral treatment with mangiferin at both doses of 10 and 20 mg/kg comparatively inhibited 3-NP triggered MDA elevation.

#### 3.4.3 GSH

GSH, a tripeptide composed of cysteine, glutamate and glycerine, is a potent endogenous non-enzymatic antioxidant involved in scavenging free radicals at the cellular level (Zitka et al., 2012). In the present study, there was a significant diminution of GSH level in the rat brain regions of the hippocampus, striatum and cortex in 3-NP treated rats as compared to the normal control group (*p* < 0.01) (Figure 7C). Interestingly, the reduced level of GSH in all three brain regions was markedly restored towards normal by mangiferin treatments (10 and 20 mg/kg, p. o.) as compared to 3-NP alone treated group (*p* < 0.05).

#### 3.4.4 SOD

SOD is a group of metalloenzymes. It acts as an enzymatic antioxidant which confers defence against cellular oxidative stress by catalysing the dismutation of superoxide anion to hydrogen peroxide (Younus, 2018). 3-NP treated rats exhibited decreased SOD activity in the hippocampus, striatum and cortex compared to normal control rats (*p* < 0.01) (Figure 7D). In the treatment groups, mangiferin administration (10 and 20 mg/kg, p. o.) significantly enhanced the activity of SOD in these brain regions when compared to 3-NP alone treated group (*p* < 0.05).

#### 3.4.5 CAT

CAT, also known as an enzymatic antioxidant, play a vital role in completing the detoxification process along with SOD. It catalyses the degradation of hydrogen peroxide (Ighodaro and Akinloye, 2018). In this study, 3-NP treatment (15 mg/kg, i. p.) resulted in a marked reduction in CAT activity in the rat brain regions of the hippocampus, striatum and cortex as compared to the normal control group (*p* < 0.01) (Figure 7E). In contrast, mangiferin treatments (10 and 20 mg/kg, p. o.) considerably protected the brain against 3-NP induced reduction of CAT activity with an evidential increase in CAT activity in all three brain regions as compared to 3-NP alone treated group (*p* < 0.05).

### 3.5 Effect of mangiferin on pro-inflammatory markers

**FIGURE 8:**
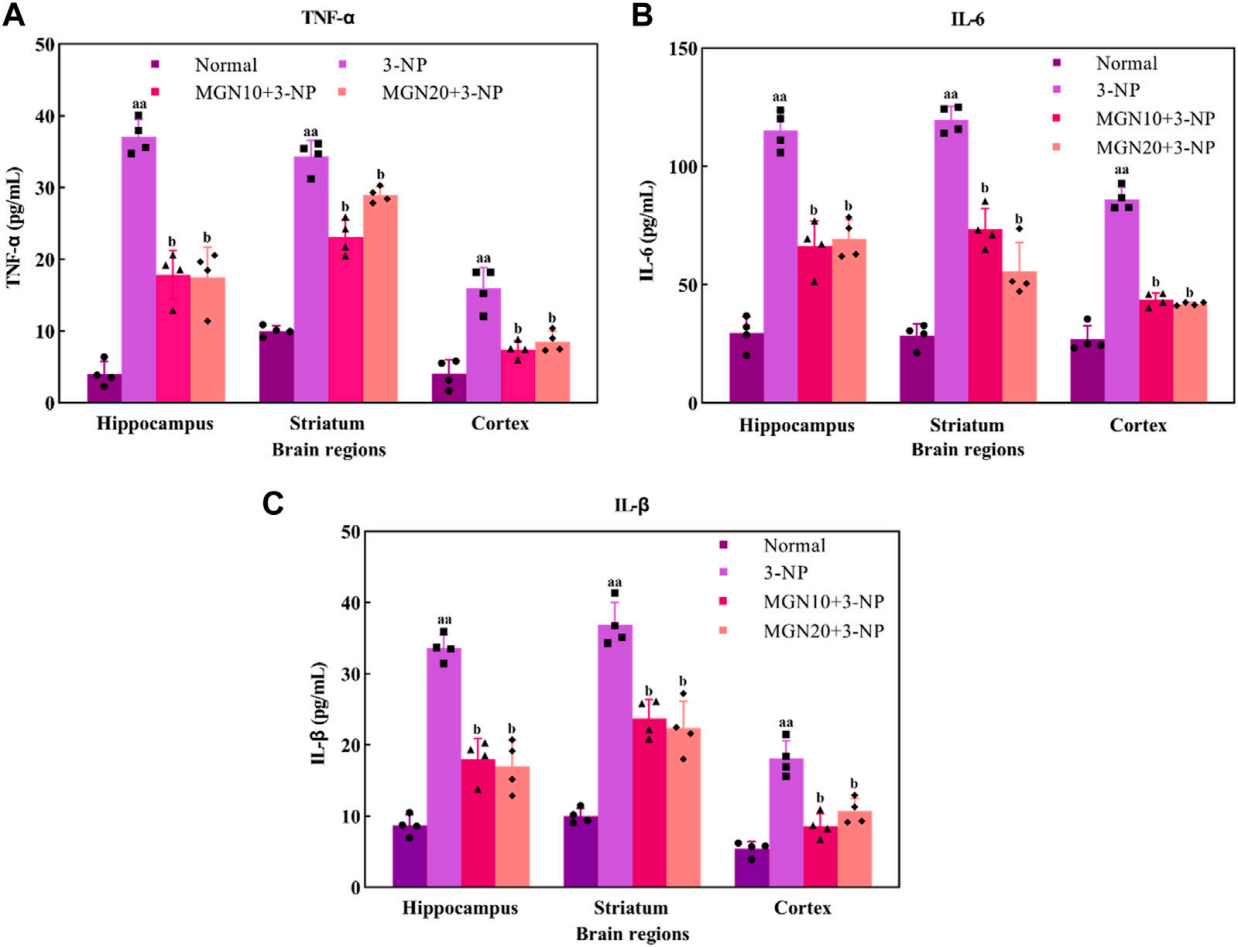
Effect of mangiferin on pro-inflammatory markers: **(A)** TNF-α, **(B)** IL-6 and **(C)** IL-1β level. Data represents mean ± SEM, *n* = 4 per group. ^aa^ *p* < 0.01 compared to normal control group; ^b^ *p* < 0.05 compared to 3-NP alone treated group.

Pro-inflammatory cytokines, such as TNF-α, IL-6 and IL-1β, play a crucial role in the pathophysiology of neurodegenerative diseases. TNF-α, predominantly secreted by activated macrophages, B lymphocytes and natural killer cells, is emerged as a key marker with major regulation in inflammatory responses (Kany et al., 2019) (Figure 8A). An increased level of TNF-α in the hippocampus, striatum and cortex was observed in 3-NP treated rats compared to the normal control group (*p* < 0.01). On the contrary, 3-NP triggered elevation of TNF-α in these brain regions was significantly averted with mangiferin treatments (10 and 20 mg/kg, p. o.) when compared to 3-NP alone treated group (*p* < 0.05).

IL-6, one of the inflammatory cytokines synthesized by macrophages and T lymphocytes, is mainly involved in mediating of inflammation and immune response (Santa Cruz et al., 2021). In particular, 3-NP administration (15 mg/kg, i. p.) induced a higher level of IL-6 in the brain regions of the hippocampus, striatum and cortex as compared to the normal control group (*p* < 0.01) (Figure 8B). Apparently, mangiferin treatments (10 and 20 mg/kg, p. o.) were shown to prevent the raise in the brain IL-6 level as compared to 3-NP alone treated group (*p* < 0.05).

IL-1β is also an inflammatory mediator from the interleukin-1 family produced by macrophages, monocytes, B lymphocytes, natural killer cells, neutrophils and dendritic cells (DC) (Garlanda and Jaillon, 2016). Similar to TNF-α and IL-6, there was a marked elevation of IL-1β level in the hippocampus, striatum and cortex in 3-NP treated rats when compared to the normal control group (*p* < 0.01) (Figure 8C). Importantly, the level of IL-1β was considerably decreased with mangiferin oral treatments at the doses of 10 and 20 mg/kg when compared to the 3-NP alone treated group (*p* < 0.05).

### 3.6 Histopathological observation

**FIGURE 9:**
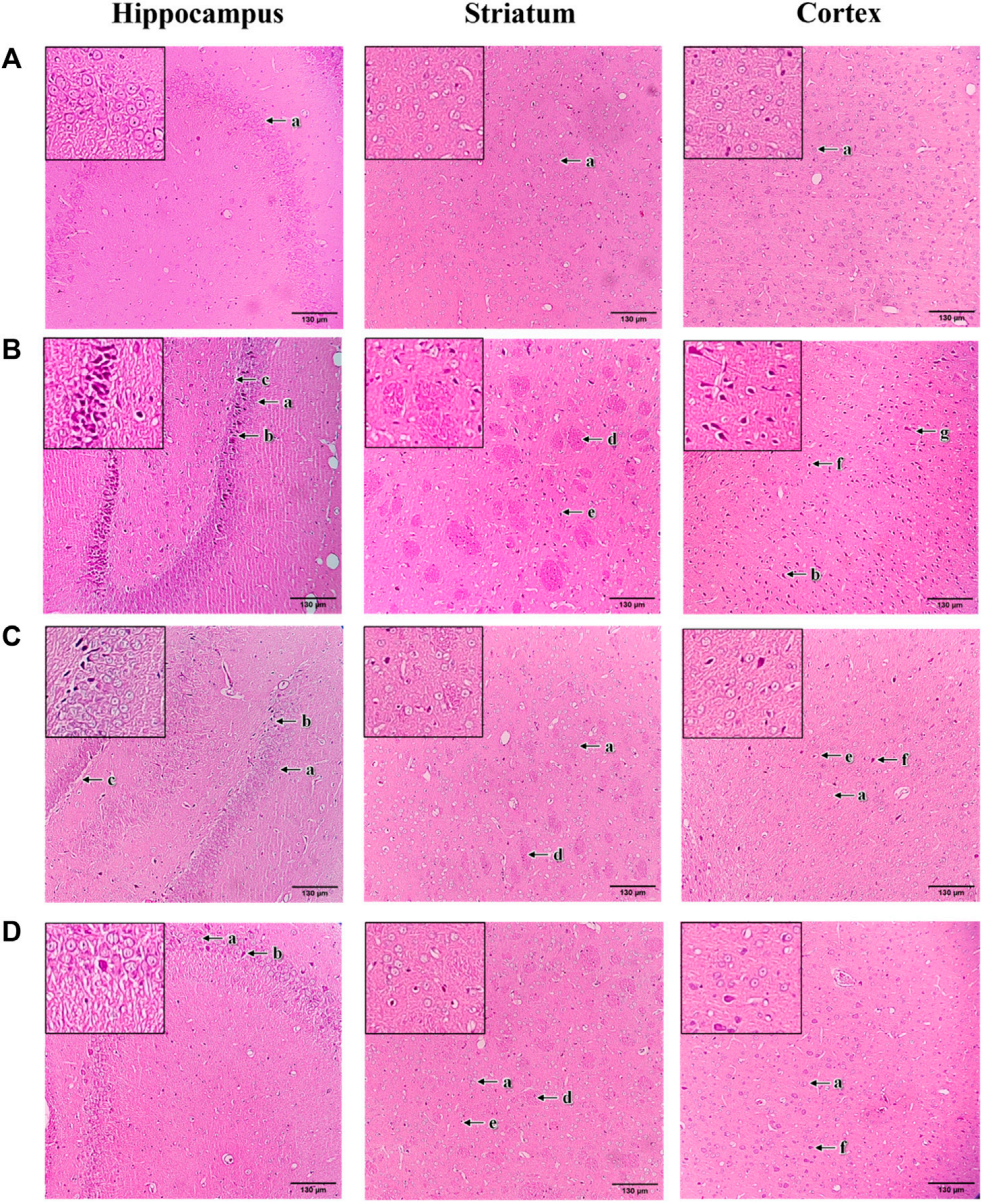
Photomicrographs of H&E-stained brain sections of hippocampus, striatum, and cortex with a magnification of ×100 for **(A)** normal control, **(B)** 3-NP, **(C)** mangiferin (10 mg/kg) + 3-NP, and **(D)** mangiferin (20 mg/kg) + 3-NP groups. a, Normal pyramidal cells; b, Necrotic pyramidal cells; c, Intercellular oedema; d, Excess degenerative plaque; e, Pyknotic neuronal cells; f, Neuronal degeneration; and g, Shrunken and necrotic neuronal cells with neurofilbrillary tangles.

The histopathological changes of brain tissue in the hippocampus, striatum and cortex sections of rats were examined under a light microscope with a magnification of ×100 (Figure 9). The histopathological observation in the brain hippocampus, striatum and cortex of normal control rats showed normal histological structure with undamaged neuronal cells, observable cell nuclei and intact cell membrane (Figure 9A). Compared to the normal control group, these brain sections in 3-NP treated rats significantly exhibited an increase in damaged neuronal cells, irregular and dense pyknotic nuclei along with marked cell necrosis (Figure 9B). Also, excess degenerative plaques and cell shrinkage with degenerated neurofibrillary were observed in the striatum and cortex regions, respectively, indicating massive neuronal degeneration caused by 3-NP (Figure 9B). However, mangiferin treatment (10 and 20 mg/kg, p. o.) remarkably mitigated 3-NP induced histopathological alterations, as evident from the mild neuronal degeneration accompanied by observable reduced pyknotic and necrotic cells in the brain sections of hippocampus, striatum and cortex, as compared to the 3-NP treated group (Figures 9C,D). Moreover, reduced degenerative plaques in the striatum region were observed, suggesting less striatal damage in both mangiferin treated groups.

## 4 Discussion

This study demonstrated that mangiferin (1) alleviated 3-NP induced body weight changes (increased body weight gain), behavioural deficits (improved anxiety-like behaviour, recognition memory, locomotor activity, neurological scoring, rotarod performance and grip strength), (2) restored 3-NP induced biochemical alteration (decreased MDA level, increased level of GSH, enhanced SDH, SOD and CAT activities), and (3) mitigated 3-NP induced histopathological changes (mild neuronal degeneration) in rats. 3-NP, an irreversible inhibitor of SDH, effectively produces HD-like symptoms such as striatal degeneration along with cognitive and motor abnormalities in rat models. Although the underlying mechanism of action of 3-NP is not clearly understood, but oxidative stress-driven mechanisms, including mitochondrial dysfunction, neuroinflammation, and excitotoxicity were proposed (Singh et al., 2015; Bhangale et al., 2016; Mehan et al., 2018). Therefore, 3-NP induced model was used to investigate the neuroprotective efficacy of mangiferin in HD *in vivo* model. In this regard, 3-NP sub-chronic administration at 15 mg/kg/day, i. p. for 7 days, substantially caused poor body weight gain in rats. The present finding is similar to previous literature as rats treated with 3-NP triggered a reduction in body weight (Dhadde et al., 2016; Karandikar and Thangarajan, 2017). These results could be attributed to 3-NP induced mitochondrial dysfunction following depressed cellular energy metabolism (Binawade and Jagtap, 2013) and sustainable metabolic alteration in the brain (Túnez et al., 2010), mimicking consistent features in the initial phase of HD patients. Accordingly, 3-NP irreversibly inhibits SDH activity in the Krebs cycle and electron transport chain (Malik et al., 2022), simultaneously interrupts the activities of mitochondrial complexes along with the loss of membrane potential and mitochondrial dysfunction, which ultimately causes adenosine triphosphate (ATP) depletion and impaired energy metabolism (Túnez et al., 2010).

Intriguingly, mangiferin treatment for 14 days markedly prevented poor weight gain in 3-NP treated rats, indicating the restoration of SDH activity and mitochondrial function by mangiferin owing to its antioxidant effects. Recovered energy metabolism sequentially improved weight gain in rats. This finding is evidenced by the biochemical data showing that mangiferin administration reversed the declined SDH activity in 3-NP treated rats. An emerging study supports the investigation that mangiferin recovered isoproterenol-induced body weight changes towards the normal range in heart failure rats (Jiang et al., 2020). Besides, the report suggests that mangiferin exerted regulatory effects on body weight in streptozotocin-induced diabetic animal models (Wu et al., 2021).

In agreement with the previous findings (Danduga et al., 2018; Sharma et al., 2021; Salman et al., 2022), in this study, memory and cognitive impairment were encountered by 3-NP treated rats. The rats revealed higher anxiety and hampered recognition memory with decreased recognition index following 3-NP administration. It has been postulated that the memory and cognitive deficits caused by 3-NP are mainly ascribed to the neuronal loss in the hippocampal regions CA1 and CA3 (Danduga et al., 2018), which is critical for episodic and spatial memory (Dimsdale-Zucker et al., 2018). 3-NP intoxicated anxiety was due to dysfunction of striatal neurons (Sharma et al., 2021). Also, 3-NP was found to produce lesions specifically in the brain regions involving cognition, including the hippocampus, striatum and cortex (Dhadde et al., 2016). In this context, accumulating evidence suggests that 3-NP induced oxidative stress was considered a culprit for brain lesions and neuronal loss in HD (Túnez et al., 2010). The cognitive disturbances in HD are said to be subcortical dementia due to the nature of brain pathology involving striatal−subcortical pathways (Nopoulos, 2022). As observed clinically in HD patients, cognitive abilities decline progressively for years before the diagnosable motor onset of HD (Bates et al., 2015). In contrast to memory storage problems, HD patients are more likely to have slow recognition and trouble with memory retrieval (Bates et al., 2015; Ross and Margolis, 2022).

Along with cognitive deficits, 3-NP intoxicated rats exhibited motor abnormalities which were showed by reduced locomotor activity, shorter latency to fall from rotarod, and decreased grip strength. Partial limb reflex of rats was also observed in 3-NP alone treated group, as an indicator of neurological dysfunction (Davuljigari et al., 2021). These results are in tune with the previous studies (Thangarajan et al., 2014; Karandikar and Thangarajan, 2017; Moghaddam et al., 2021) as 3-NP caused deficiencies in locomotor and motor function by affecting the striatum, which is essential for body movement control (Kaur et al., 2016). The abnormalities were associated with marked neurodegeneration in striatum (Chakraborty et al., 2014) and cell gliosis (Suganya and Sumathi, 2017) induced by overwhelming oxidative/nitrosative stress in 3-NP action (Sharma et al., 2021).

Importantly, mangiferin treatment significantly mitigated 3-NP triggered memory and cognitive disabilities in rats, which is evidenced by reduced anxiety with increased time spent in center and restored spatial memory with higher recognition index to the novel object. As similar in previous literature, mangiferin treated mice (20 and 40 mg/kg, p. o.) shows reduced anxiety and depression from the results of open field test, light-dark box and elevated plus maze in the lipopolysaccharide (LPS) induced model (Jangra et al., 2014). Likewise, mangiferin was shown to exert significant protection on motor coordination in 3-NP treated rats, highlighted by improved locomotor activity, enhanced rotarod performance, increased grip strength and restored neurological behaviours. These findings are in accordance with the previous studies as growing evidence implicates the potential of mangiferin in alleviating anxiety and depressive-like behaviours, memory and learning deficits, and movement deficiencies by virtue of antioxidant and anti-inflammatory properties (Feng et al., 2014).

Oxidative stress is known as a chief determinant of neurotoxicity in HD (Bates et al., 2015). In this study, 3-NP intoxication significantly disrupted energy metabolism via inhibition of SDH activity. Elevated oxidative stress was also observed in 3-NP treated rats, as evident from the increased MDA level, diminished level of GSH and reduced activities of enzymatic antioxidants such as SOD and CAT in the hippocampus, striatum and cortex regions. These results are in agreement with previous studies (Mahdy et al., 2014; Danduga et al., 2018), as 3-NP has been reported to increase the generation of hydroxyl free radicals and initiate the oxidative cascade activation in the brain (Shetty et al., 2015). It was also reported that oxidative stress mediated cell membrane damage and cell function loss lead to lipid peroxidation (Mehan et al., 2017). In regard to 3-NP induced neurotoxicity, oxidative metabolism induced by 3-NP triggers the sensitization of N-methyl-D-aspartic acid (NDMA) receptors and glutamate excitotoxicity, synergistically prompt an increase in intracellular calcium levels, resulting in further neuronal degeneration (Túnez et al., 2010). The alteration of dopamine and glutamate interactions is a great concern in HD. There is evidence that dopamine and glutamate neurotransmissions are affected in HD (André et al., 2010). In HD patients, it has been stated that abnormal extracellular dopamine levels with elevated glutamate levels lead to excitotoxicity, loss of striatal and cortical neurons, thereby develop to the later symptoms such as dystonia or akinesia (André et al., 2010).

The levels of pro-inflammatory cytokines, including TNF-α, IL-6, and IL-1β were also markedly increased in the brain regions of hippocampus, striatum and cortex for 3-NP intoxicated rats. As documented in the previous findings, 3-NP treatment indicates an increase in microglial activation and mRNA expression of TNF-α, IL-6, IL-1β, inducible NOS (iNOS) and cyclooxygenase-2 (COX-2) in the striatum (Jamwal and Kumar, 2016; Jang et al., 2019). Recent literature has reported that high level of mHtt in monocytes and microglia as well as the accumulation of activated microglia and reactive astrocytes are observed in the brain of HD patients (Valadão et al., 2020), suggesting that microglia activation is one of the major sources of cytokine modulation in HD (Palpagama et al., 2019). It has been stated that mHtt aggregate positively activates microglia and induces the release of pro-inflammatory mediators such as TNF-α, IL-6, IL-1β, and IL-8 via NF-κB signaling, with an increase of anti-inflammatory mediators such as IL-4 and IL-10 but at the later stages of HD (Tai et al., 2007; Björkqvist et al., 2008). Persistent microglia activation along with prolonged inflammatory cytokines production could lead to chronic inflammation in brain (Ellrichmann et al., 2013). Further, aggregation of mHtt in astrocytes results in reactive astrogliosis followed by glutamate excitotoxicity, contributing to HD neuronal death (Khakh et al., 2017).

In view of the above mentioned, the results from behavioural studies were further supported by the results from biochemical estimation. Oral administration of mangiferin (10 and 20 mg/kg, p. o.) in rats greatly protected the brain regions of the hippocampus, striatum and cortex against 3-NP induced oxidative stress. The SDH activity was found to be enhanced by mangiferin in 3-NP treated rats. As well, a significant decrease in MDA level along with an increased level of GSH and restored activities of SOD and CAT in the hippocampus, striatum and cortex were observed in mangiferin treated rats. The present findings are in line with the previous investigation of various natural products such as solanesol (Mehan et al., 2018), embelin (Dhadde et al., 2016), spermidine (Jamwal and Kumar, 2016), lutein (Binawade and Jagtap, 2013) and lycopene (Sandhir et al., 2010) on HD *in-vivo* models. Similarly, the study of (Kavitha et al., 2013) also reveals that mangiferin (10, 20, and 40 mg/kg, p. o.) ameliorates 1-methyl-4-phenyl-1,2,3,6-tetrahydropyridine (MPTP) induced oxidative stress with reduced brain lipid peroxidation and GSH level in Parkinson’s disease (PD) mice model. The prophylactic effect of mangiferin in reversing these alterations in biochemical parameters is due to the antioxidant activity in sequestering free radicals and enhancing brain cellular antioxidant defence (Sandhir et al., 2010; Mahdy et al., 2014). In Alzhemier’s disease (AD) *in vitro* model, it has been stated that mangiferin effectively suppress β-amyloid (Aβ) induced neurotoxicity on brain cell via scavenging of ROS (Sethiya and Mishra, 2014). Ample reports implicate that mangiferin possesses free radicals scavenging activity in neuroprotection was mainly ascribed to its nature of C-glucosy linkage along with the presence of polyhydroxy components (Walia et al., 2020; Liu et al., 2021).

Besides, mangiferin treatment was shown to attenuate the elevation in TNF-α, IL-6, and IL-1β levels in the brain hippocampus, striatum and cortex, indicating the anti-inflammatory effect of mangiferin against 3-NP induced neuroinflammation. Consistent with these results, other studies also support the working model of mangiferin on neurodegenerative diseases, in which it could ameliorate the neuroinflammatory responses by declining the expression of IL-6 and IL-1β (Feng et al., 2019). In the study of Luo et al. (2017), mangiferin treatment (40 mg/kg, p. o.) mitigated corticosterone triggered an increased level of TNF-α and IL-6 in the hippocampus. Similarly, mangiferin administration (20 and 40 mg/kg, p. o.) depleted AlCl_3_ induced elevated release of TNF-α and IL-1β (Kasbe et al., 2015). It has been postulated that mangiferin reduces the secretion of pro-inflammatory cytokines by inhibiting the activation of astrocytes and microglia via the NF-κB signaling pathway (Infante-Garcia et al., 2017). In this context, it has been reported that mangiferin suppresses microglial activation by downregulating the synthesis of COX-2 and prostaglandin E2 (PGE-2) in both LPS induced *in vitro* (Bhatia et al., 2008) and *in vivo* (Jangra et al., 2014) models, indicating the vital role of mangiferin to protect the neurons from the neuroinflammatory response.

Apart from this, the neuroprotective potential of mangiferin against 3-NP induced HD rat model was further evaluated by histopathological examination of the brain regions of the hippocampus, striatum and cortex. In concordance with previous reports (Mehan et al., 2017; Mehan et al., 2018), in this study, histopathological changes with marked neurodegeneration were produced by 3-NP in the brain hippocampus, striatum and cortex of rats. These results further affirm that 3-NP induced cognitive and motor impairments were due to neuronal dysfunction. In contrast, the promising protective effect of mangiferin was confirmed with the mild degenerative changes in the brain tissue of the hippocampus, striatum and cortex in mangiferin treated groups. Supporting the histopathological results, 3-NP induced behavioural deficits and biochemical changes were greatly improved by mangiferin in this study.

**FIGURE 10:**
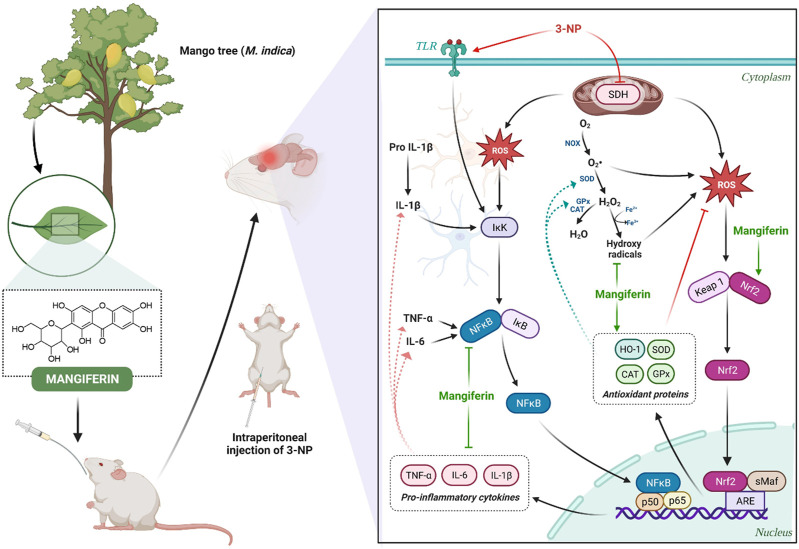
Neuroprotective mechanism of mangiferin via antioxidant and anti-inflammatory pathways against 3-NP induced HD rat model.

Mounting pieces of evidence have indicated that mangiferin exerts its antioxidant mechanism in neuroprotection by 1) sequestering free radicals (Kasi et al., 2010), 2) reversing the decreased level of cellular antioxidants (Amazzal et al., 2007), 3) attenuating the elevated level of cellular oxidative stress by neutralizing excess ROS (Feng et al., 2017). The schematic diagram representing the antioxidant and anti-inflammatory mechanism of mangiferin are depicted in Figure 10. In reacting to oxidative stress, a compensatory mechanism of the endogenous antioxidative defence system is induced by mangiferin via the activation of the Nrf2 signaling pathway. Nrf2 is a redox-sensitive transcription factor that essentially regulates the ARE-driven gene expression of antioxidant proteins (Gil and Rego, 2008). The activated Nrf2-ARE signaling pathway sequentially induces the metabolizing antioxidant enzymes in exerting antioxidant effects by upregulating the enzymatic antioxidants (SOD, CAT, and GPx) and non-enzymatic antioxidants (GSH), to neutralize the excess ROS in the brain (Li et al., 2011). Heme oxygenase 1 (HO-1), a rate-limiting enzyme with the most abundant AREs, is also regulated by Nrf2 to catalyse the oxidative degradation of heme to biliverdin, produce carbon monoxide and ferrous ion in scavenging ROS (Wang Z. et al., 2017). Taken together, lipid peroxidation is reduced and these activities can directly protect the distinctive cells and neurons against 3-NP-induced oxidative damage (Malik et al., 2017).

Accordingly, neuroinflammation also occurs in the pathological mechanism of HD. In response to the immune reactions in the central nervous system, astrocytes and microglia are activated and an increased level of pro-inflammatory cytokines (TNF-α, IL-6, and IL-1β) is secreted to impair the neurons (Stephenson et al., 2018). NF-κB is a transcription factor that plays a vital role in the mediation of cell proliferation, immunity, inflammation and survival (Liu et al., 2021). The inhibitor of NF-κB (IκB), the upstream factor of NF-κB, is activated via the phosphorylation of IκB kinase (Alam et al., 2021). Extensive studies suggest that mangiferin could inhibit the degradation of IκB and hinder the activation of NF-κB, to regulate the transcription of several inflammatory cytokines-related genes upon inflammation (Feng et al., 2019; Liu et al., 2021). Along with this, activation of astrocytes and microglia is inhibited by blocking the NF-κB pathway, thereby further attenuating the delivery of pro-inflammatory cytokines to the immune system in HD (Fu et al., 2014).

## 5 Conclusion

**FIGURE 11:**
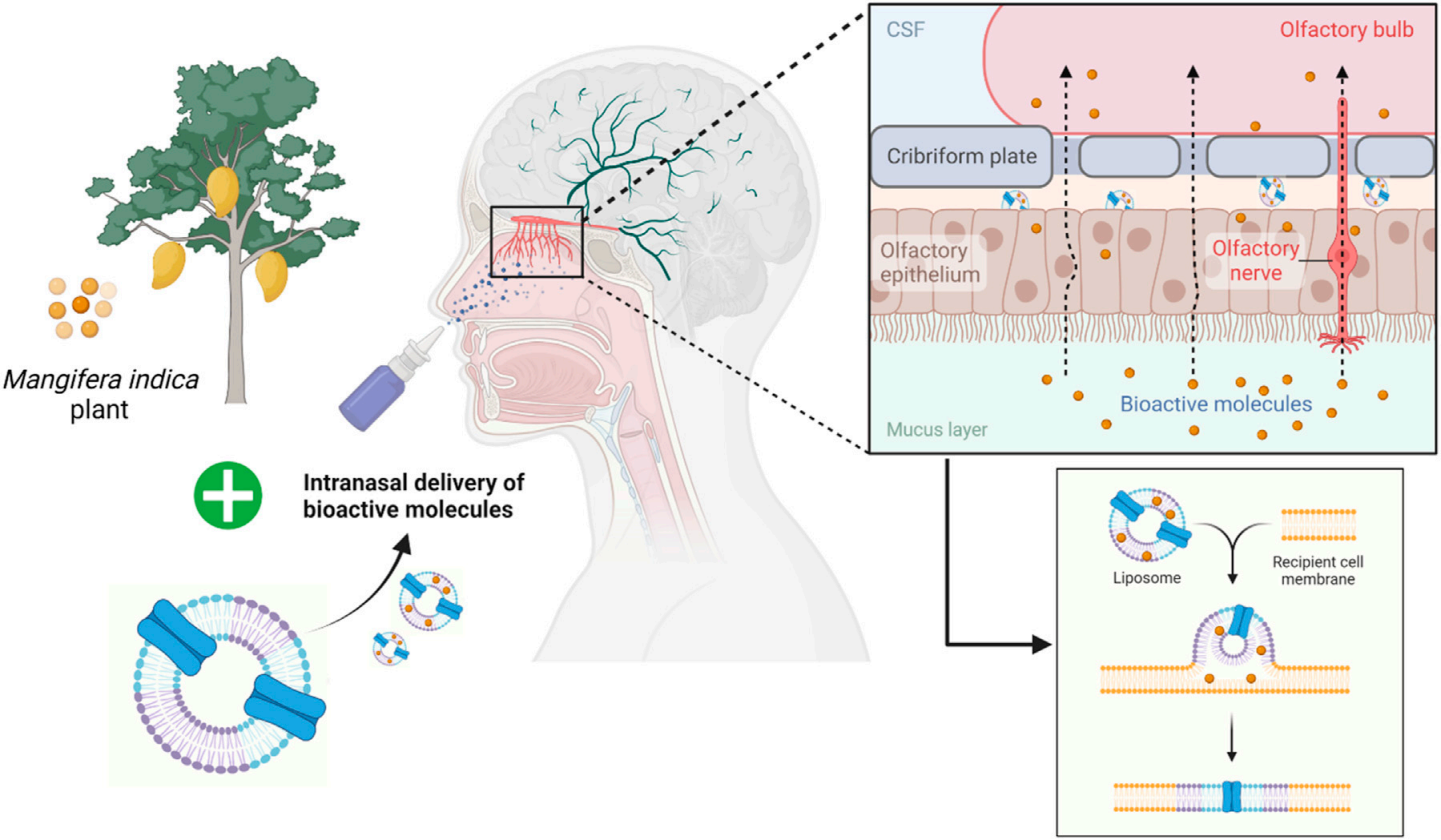
Future prospective of employing liposomes as an intranasal delivery of bioactive molecules across the olfactory bulb.

In conclusion, the present study indicated the neurotherapeutic potential of mangiferin against 3-NP induced body weight, behavioural, biochemical and histopathological alteration in rats. This provides a new insight into the working model of mangiferin as a neuroprotective agent for 3-NP induced HD-like symptoms by virtue of antioxidant and anti-inflammatory activities. Mangiferin may replace conventional medicines in the treatment and prevention of HD due to its excellent safety profile. However, human research findings are insufficient. Thus, further dedicated works on clinical efficacy are recommended to envisage to its short- or long-term medicinal uses for improvement in HD. In the future, mangiferin can also be incorporated into nanocarriers such as liposomes and given intranasally (Figure 11). The liposomes encapsulating the bioactive compound will be exocytosed in the olfactory bulb after being endocytosed by the olfactory sensory cells. Then, the olfactory neurons replicate this trans-synaptic process, transferring the biomolecules to different parts of the brain to effectively reduce the progression of the disease.

## Data Availability

The raw data supporting the conclusion of this article will be made available by the authors, without undue reservation.
