# Supplementary material for: Neuroprotective potency of mangiferin against 3-nitropropionic acid induced Huntington’s disease-like symptoms in rats: possible antioxidant and anti-inflammatory mechanisms
Source: Front Pharmacol. 2023 Jul 13;14:1189957. doi: 10.3389/fphar.2023.1189957 (PMC10372348; doi:10.3389/fphar.2023.1189957)
Supplement: Supplementary file 1 [file Table1.DOCX]

Supplementary Material

Neuroprotective Potential of Mangiferin Against 3-Nitropropionic Acid Induced Huntington’s Disease-Like Symptoms in Rats: Possible Antioxidant and Anti-inflammatory Mechanisms

Pei Teng Lum^1^, Mahendran Sekar^2,1*^, Lay Jing Seow^1*^, Mohd. Farooq Shaikh^3,4^, Alina Arulsamy^4^, Thaarvena Retinasamy^4^, Siew Hua Gan^2^, Charles Gnanaraj^5^, Norhaizan Mohd Esa^6^, Gobinath Ramachawolran^7*^, Vetriselvan Subramaniyan^8^, Suresh V. Chinni^9,10^ and Yuan Seng Wu^11^

*** Correspondence:** Mahendran Sekar: [mahendran.sekar@monash.edu](mailto:mahendran.sekar@monash.edu) ; Lay Jing Seow: [ljseow@unikl.edu.my](mailto:ljseow@unikl.edu.my); Gobinath Ramachawolran: [r.gobinath@rcsiucd.edu.my](mailto:r.gobinath@rcsiucd.edu.my)

# Supplementary Figures


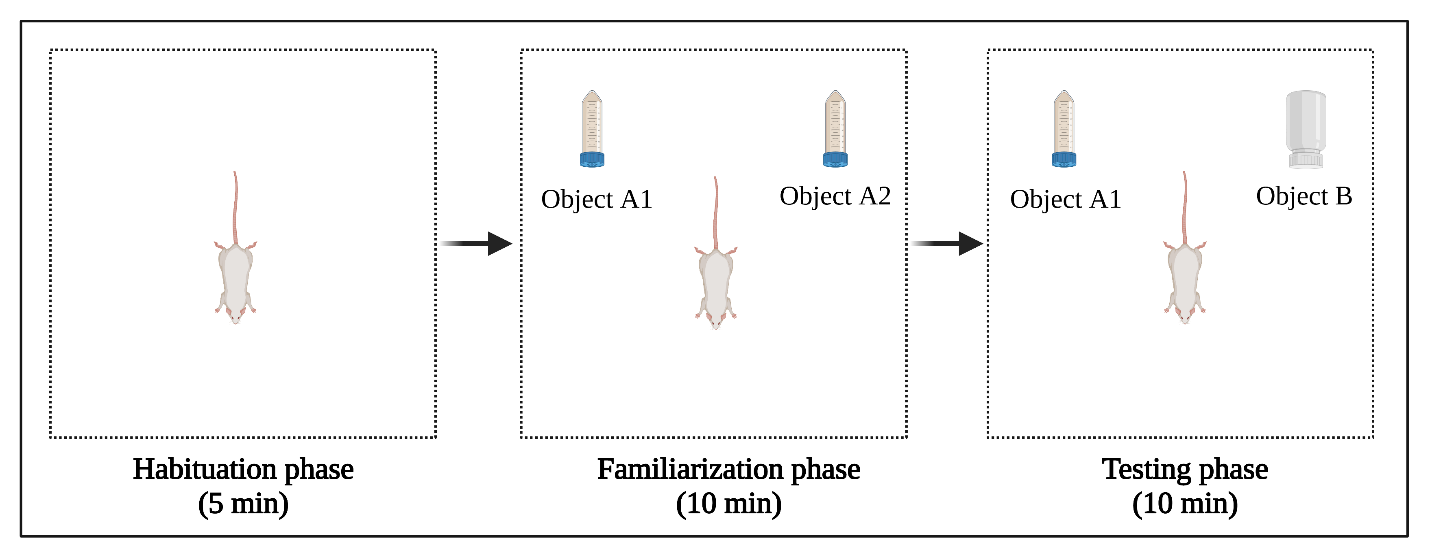


**Supplementary Figure 1.** Novel object recognition experimental protocol.


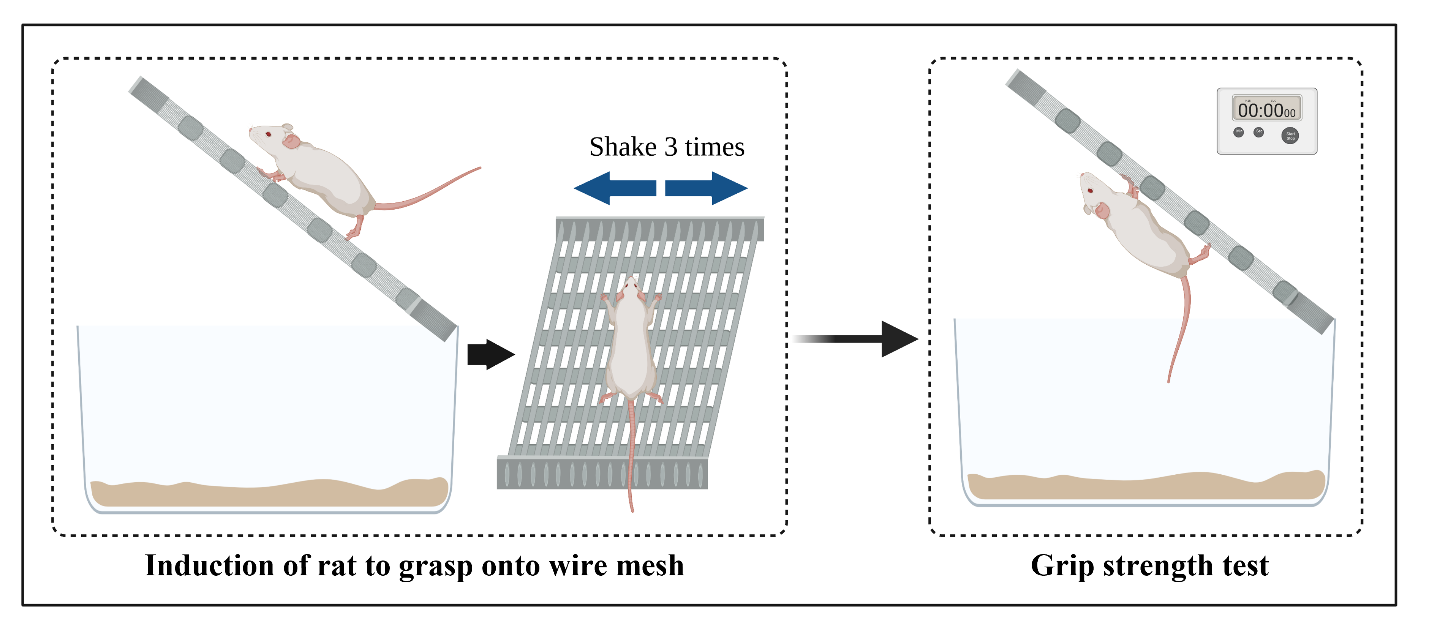


**Supplementary Figure 2.** Schematic illustration of grip strength test.
